# Adversarial Patch Attacks on Deep-Learning-Based Face Recognition Systems Using Generative Adversarial Networks

**DOI:** 10.3390/s23020853

**Published:** 2023-01-11

**Authors:** Ren-Hung Hwang, Jia-You Lin, Sun-Ying Hsieh, Hsuan-Yu Lin, Chia-Liang Lin

**Affiliations:** 1 College of Artificial Intelligence, National Yang Ming Chiao Tung University, Tainan 71150, Taiwan; 2 Computer Science and Information Engineering Department, National Chung Cheng University, Chiayi 62102, Taiwan; 3 Telecom Technology Center, Kao-Hsiung 82151, Taiwan

**Keywords:** deep learning, face recognition, adversarial attack, perturbation, adversarial examples, adversarial patches, Generative Adversarial Network

## Abstract

Deep learning technology has developed rapidly in recent years and has been successfully applied in many fields, including face recognition. Face recognition is used in many scenarios nowadays, including security control systems, access control management, health and safety management, employee attendance monitoring, automatic border control, and face scan payment. However, deep learning models are vulnerable to adversarial attacks conducted by perturbing probe images to generate adversarial examples, or using adversarial patches to generate well-designed perturbations in specific regions of the image. Most previous studies on adversarial attacks assume that the attacker hacks into the system and knows the architecture and parameters behind the deep learning model. In other words, the attacked model is a white box. However, this scenario is unrepresentative of most real-world adversarial attacks. Consequently, the present study assumes the face recognition system to be a black box, over which the attacker has no control. A Generative Adversarial Network method is proposed for generating adversarial patches to carry out dodging and impersonation attacks on the targeted face recognition system. The experimental results show that the proposed method yields a higher attack success rate than previous works.

## 1. Introduction

Face recognition technology has undergone significant advances in recent years through the application of deep learning models. Meanwhile, the COVID-19 pandemic has brought about many lifestyle changes, including a desire for non-contact business opportunities wherever possible [1]. As a result, face recognition now plays a significant role in improving security and convenience in all manner of fields and applications. For example, face recognition is widely used throughout manufacturing and warehousing, banking and financial insurance, smart offices, smart homes, retail, public transportation and airports, medical scenes, schools and education institutions, hotels, and many other service industries. MarketsandMarkets [2] estimated that the global output value of face recognition would grow at an annual rate of 17.2% from 2020 onwards and would reach a global market value of USD 13.87 billion in 2028. Thus, face recognition offers significant business opportunities in the coming years and decades.

However, despite the many benefits of deep learning technology, it is not without risk. For example, the authors in [3] showed that image classification systems built on deep learning models can be easily attacked by adding a small perturbation to the original image to form an adversarial example, which is subsequently misclassified. Similar misclassification errors can be induced by applying adversarial patches [4] to the original image to produce local perturbations. In such cases, the reliability of the image classification system is significantly impaired. Consequently, the problem of improving the robustness of deep learning models, and the applications which rely on these models (face recognition systems, for example), is a crucial concern in real-world environments.

Based on the above issue, we propose a method that can make the face recognition model misclassified, and the method can achieve attack effectiveness in the physical world as well. Moreover, we also explore adding adversarial images to the face recognition model as a training dataset to improve the model’s robustness.

Adversarial attacks against deep learning models can be divided into many types. For example, depending on the adversarial capacity, they can be classified as either white-box or black-box attacks, where in the former case, the attacker knows the parameters and architecture of the deep learning model, whereas in the latter case, they do not. Black-box attacks are thus generally more challenging than white box attacks. A second class of attack is that of poisoning attacks, in which adversarial images are injected into the model during the training stage in order to affect the learning performance; or input or evasion attacks, in which the input images are deliberately perturbed in order to produce misclassification errors. Depending on the space in which they are launched, adversarial attacks can also be classified as either physical world attacks [5,6] or digital world attacks. Finally, depending on whether or not the attack has a specific target, adversarial attacks can be categorized as either targeted attacks or non-targeted attacks, where such attacks are generally referred to as dodging attacks or impersonation attacks, respectively, in the face recognition field. Dodging attacks aim to cause the input face image to be identified as any other individual in the face database. By contrast, impersonation attacks aim to cause the individual to be identified as a specific person (i.e., the attack target) in the database.

However, for the attack method, Bhambri et al. [7] surveyed the relevant literature, in which Deb et al. [8] proposed a GAN-based [9] adversarial attack method that generates perturbations for the human face to achieve an attack in a digital environment. That perturbation cannot be examined with the human eye, nor can physical cameras. Therefore, based on this method, we can conduct an attack on a face recognition system in the physical world by generating perturbation for the glasses of a specific person. When an attacker wears attack glasses to attack a face recognition system, it can cause misidentification.

As aforementioned, the majority of face recognition systems are built in the real world, the present study focuses on the challenging problem of black-box input attacks using GAN-based adversarial patches in the physical world. For the sake of robustness, the study considers both dodging attacks and impersonation attacks. In short, our contributions are summarized as follows.

- We propose the adversarial patches method for face recognition attacks applicable to the physical world. It does not require knowledge of the parameters of the deep learning model (black box) to achieve attack effectiveness.
- For the reliability of our approach, we performed a comprehensive attack test for all one-to-one combinations. Based on testing quantities, the number of subjects and the number of testing by each subject is higher than the previous literature, which results show that the success rate of dodging attacks is 57.99%, and the impersonation attack success rate is 46.78% in the digital world. The success rate of dodging attacks is 81.77%, impersonation attack success rate reached 63.85% in the physical world.
- The proposed attack method utilizes the adversarial patch, which occupies only a small area of the face, instead of the adversarial example, which occupies the whole face. Therefore, the attacker can adjust the noise region according to the requirements. In our case, we hide the adversarial perturbation in the glasses to achieve the effectiveness of being judged as someone else. As a result, it is difficult for the layperson to know our attack intent and, therefore, poses a significant threat to the face recognition system.
- Based on the method proposed in this study, we found that the number of people with two face databases of different numbers of people, the number of people will further affect the attack’s success rate. The attack success rate increases when the number of people in the database increases. In short, the chances of being hacked increase.
- We propose a novel defense mechanism to counter the GAN-based adversarial patch method. The results show that the proposed mechanism can detect almost all dodging attacks and more than half of the impersonation attacks with high defense effectiveness.
- We explored the relationship between thresholds and attack success and proved that both are relative. In addition, we attack different models by the no-box attack, showing that our attack method is transferable.

## 2. Background

### 2.1. Face Recognition

Various face recognition methods have been proposed in the past, such as SVM-based, subspace learning-based, and deep-learning-based methods. We summarize and compare the previous works in Table 1.

In many classification issues, the samples of one class are usually surrounded by the samples of the other classes. To address this issue, Ye et al. [10] proposed multiview robust double-sided twin SVM(MvRDTSVM) and a fast version of MvRDTSVM (named MvFRDTSVM). In the Tufts face database, MvRDTSVM and MvFRDTSVM achieved an accuracy of 91.55% and 88.82%, respectively. The previous method used in the two classification problems is not suited for face recognition that has many people. In general, face recognition algorithms consist of three steps: pre-processing images, feature extraction, and face classification. Lahaw et al. [11] proposed combining Two Dimensional Discrete Wavelet Transform (2D-DWT), which can capture localized information of images, with Principal Component Analysis (PCA), Linear Discriminant Analysis (LDA), or Independent Component Analysis (ICA) to extract face feature. Finally, the SVM algorithm combined with the 2D-DWT method has led to the increase of the performance of PCA + SVM, LDA + SVM, and ICA + SVM from 90.24%, 93.9%, and 91% to 96%, 96%, and 94.5%, respectively. Most of the existing distance metric-based(DML) methods are *k*NN DML methods. The disadvantage of *k*NN DML method is that the classification result is affected by the setting of the nearest neighbor number *k*. Ruan et al. [12] proposed a convex model for support vector DML (CSV-DML), which increased the accuracy of the CSV-DML to 84.6%, better than the existing *k*NN DML and support vector DML methods.

Furthermore, many well-known face recognition studies are based on deep learning approaches, including Deepface [13], FaceNet [14], VGG-Face [15], and ArcFace [23]. Deepface [13], proposed by Facebook in 2014, uses a nine-layer neural network with Softmax in the loss function, and achieved a recognition accuracy of 97.35% when applied to the LFW (Labeled Faces in the Wild) dataset. FaceNet [14] was proposed by Google in 2015 and uses ZFNet and Inception-v1 as the Siamese network [24] architecture and a triplet loss in the loss function. The model achieved an accuracy of 99.63% on the LFW dataset in the validation stage. The Visual Geometry Group (VGG) proposed VGG-Face [15] in 2017, which is a neural network for large-scale image recognition based on a small number of VGG [25] training samples and the Softmax loss function. It was shown that the accuracy of the proposed network reached 99.6% when using the triplet loss proposed in FaceNet [14] for inference purposes.

ArcFace [23], proposed in 2018, is based on the ResNet deep neural network architecture [26], but employs a novel loss referred to as Additive Angular Margin Loss. The model achieved an accuracy of 99.83% when applied to the LFW dataset.

In recent studies, Fuad et al. [27] surveyed many deep learning (DL) methods for face recognition (FR). The authors explored them in several parts. For the CNN-based method, Chen et al. [16] considered the angle discrepancy and magnitude gap between high-resolution and corresponding low-resolution faces. It successfully identified faces with fewer than 32 × 32 pixels, resulting in LightCNN-v29 achieving a 98.98% success rate. Wang et al. [17] proposed a pyramid-diverse attention framework to avoid the model focusing on fixed blocks by extracting features in multiple layers so that the model can extract facial features more comprehensively. For the Autoencoder-based method, Autoencoder combines generated and learned properties, but it still learns irrelevant features. Therefore, Pidhorskyi et al. [28] proposed an Adversarial Latent Autoencoder (ALAE) to solve this issue and improve the training procedure of GAN. Additionally, Usman et al. [18] used multiple levels of hidden layers for feature extraction and dimension reduction for expression recognition. For GAN-based methods, Iranmanesh et al. [19] proposed the CpGAN method, which processes visible and non-visible spectra separately through two sub-networks of independent GANs. The CpGAN was then used for heterogeneous face recognition. In addition, Rong et al. [20] used GAN to solve the issue of failing recognition when the identified person has a large pose change. For the Reinforcement Learning-based method, Liu et al. [21] and Rao et al. [22] applied reinforcement learning to find the attention of videos in a heterogeneous collection of unordered images and videos, and both achieve rich results.

To sum up the above methods, despite the many novel architectures proposed in the recent literature, FaceNet has a unique architecture and employs a triplet loss to process the data. FaceNet continues to be one of the most commonly used and accurate models for face recognition purposes. Although the accuracy of ArcFace is slightly higher than that of FaceNet, its performance advantage is obtained at the expense of a higher computational cost. Consequently, the present study deliberately adopts FaceNet to build the face recognition systems used for evaluation purposes.

### 2.2. Adversarial Attack

#### 2.2.1. Adversarial Example

Adversarial examples are produced by adding small perturbations to the original input sample. Many methods are available for generating adversarial examples, including the Fast Gradient Sign Method (FGSM) [3], the Basic Iterative Method (BIM) [5], the Projected Gradient Descent (PGD) [29], and the Carlini & Wagner attack [30]. All of these methods have a high attack success rate and are widely used in digital attack scenarios. However, FGSM is not suitable for black-box attacks since they require knowledge of the model parameters when training. BIM and PGD are based on FGSM, albeit with a smaller step size, and is thus equally inapplicable to black-box attacks.

Consequently, among these methods, only the Carlini and Wagner attack model can be applied to black-box attacks. Most attack methods are based on loss functions and add a gradient value to the image pixels as noise. The target model is then queried repeatedly until model convergence. However, face recognition systems are generally implemented in the real world and, provided that the face recognition system is not hacked, the likelihood of an attack succeeding simply by directly modifying the image pixels is rather low. Furthermore, if the attack queries the model many times in an attempt to deceive the system, it is likely to trigger a security mechanism and will thus similarly fail. In other words, adversarial attack methods, which add noise to the entire image, have only a limited effectiveness against face recognition applications.

#### 2.2.2. Adversarial Patch

Adversarial patches [4] differ from adversarial examples in that they add noise only to certain regions of the image, rather than the entire image. Adversarial patch attacks can be easily applied in the real world and require no knowledge of the parameters or architecture of the model. The authors in [31] demonstrated the feasibility for fooling automated surveillance cameras by applying adversarial T-shirts to the subject. Similarly, the authors in [32] generated adversarial patches for road signs using a GAN-based [9] method and showed that the patches prevented the classifier from identifying the road signs correctly.

### 2.3. Attention Area of Face Recognition

The accuracy of face recognition systems based on deep learning methods is significantly higher than that of earlier image-processing-based or statistical methods. However, besides the prediction accuracy of such methods, there is growing interest in the interpretability of the prediction results. Castanon and Bryne [33] used a heat map to quantify the relative importance of each feature in the classification model. The results indicated that the prediction outcome was determined mainly by the features extracted from the eyes, nose and mouth regions of the image. Deb et al. [8] also showed that the success rate of adversarial patch attacks against face images was enhanced when applying noise mainly to the eyes and nose regions of the face.

## 3. Related Works

The literature contains many studies on attack methods against deep-learning-based face recognition systems. However, many of these studies assume that the attacker somehow gains access to the face recognition system, or consider only attacks in the digital world. By contrast, the present study aims to explore a more realistic attack scenario, in which the attack occurs in the physical world and the attacker has no information of the model parameters and architectures. Thus, in considering the previous work in the field, the present study focuses mainly on the works shown in Table 2 where we distinguish related works by attack method, attack situation, generate object and attack capacity.

As shown in Table 2, many of the adversarial attack methods reported in the literature are white-box attacks and use adversarial patches. For example, the method in [34] uses the L-BFGS method to print 2D or 3D images with adversarial glasses. The attack success rate was found to be as much as 97.22% in dodging attacks and 75% in impersonation attacks. However, the test dataset was limited to just three individuals. In [35], an LED is added to the hat, an infrared light is projected onto the face which is adjusted according to the attack target. It is noticed that the attack is unstable which indicates some limitation on using infrared light attack. Moreover, the attack method is white-box attack. The studies in [36,37] conduct white-box attacks using adversarial stickers attached to hats and the nose, forehead and eyes regions of the face image, respectively. It was shown in [36], that the adversarial patches effectively reduce the similarity between the input image and the target image and therefore is able to attack the face recognition system. In [37], although the similarity of the patch face image with the ground truth class was only just slightly lower that that with the targeted class, the attack is successful as the neural network classifies the patch face image as the targeted class.

Although the methods in [34,35,36,37] are capable of deceiving the face recognition model, their success rate is relatively low. Moreover, the adversarial attacks are launched using white-box attack methods. As described above, white-box attacks require a knowledge of the parameters and architecture of the face recognition model. Thus, white-box attacks are generally ineffective in real-world scenarios, where such information is carefully guarded. Accordingly, the authors in [38,39] proposed black-box attacks, in which light produced by a projector was used to generate attack noise. The study in [38] attacked the FaceNet face recognition system and achieved an average dodging success rate of 85.7% for nine test subjects and an average impersonation success rate of 32.4%. In [39], projected light was generated as noise using an update gradient method and was used to conduct attacks against a commercial face recognition model. The dodging attack and impersonation attack success rates were shown to be 70% and 60%, respectively. However, the impersonation attack considered only one test subject. The attack success rates of the methods in [38,39] are generally higher than those of previous methods. However, in both cases, it is necessary to query the system multiple times during training. Moreover, it is impractical to carry and use the light projection equipment in real-world situations, and the projection angle and light intensity must be carefully considered and managed.

Deb et al. [8] used a GAN to generate adversarial noise, which was added to the original face to form an adversarial face in the digital world. In the original GAN architecture, the aim is to generate an image which is as similar to the original image as possible. In [8], however, the performance of the GAN in generating adversarial noise was improved by extending the loss function to include not only the original loss $L_{GAN}$, but also two new losses, namely $L_{perturbation}$ and $L_{identity}$, respectively. The aim of the $L_{perturbation}$ loss was to control the amount of noise generated, while that of the $L_{identity}$ loss was to control the generated noise such that the image was classified into a specified class. The GAN model was used against the FaceNet face recognition model and achieved a success rate of 97.22% for obfuscation attacks and 24.30% for impersonation attacks. Thus, even though the attack model considered the more realistic scenario of a black-box attack, the impersonation success rate was still rather low. Bin et al. [40] used a GAN-based method to add makeup around the eyes as adversarial noise. The experimental results showed that the proposed method achieved an average success rate of 33.17% for impersonation attacks against FaceNet and a maximum success rate of 52.92% for impersonation attacks against a commercial face recognition model. Xiao et al. [41] proposed another GAN-based method (advGAN) for generating adversarial examples. However, it was limited only to attacks in the digital world and was aimed at image classification systems rather than face recognition systems. Tong et al. [42] proposed the FACESEC method based on gradient $l_{0}$-norm to generate stickers, eyeglass frames, and face masks, which in turn attack FaceNet and VGGFACE. The attack success rate of the eyeglass frame on the FaceNet model is 54%. In addition, [42] explored the effect of knowing the parameter and architecture of the attack model on the attack success rate. Shen et al. [43] proposed a GAN-based adversarial attack to generate stickers that can be adhered to the ciliary arches, nasal bones, and two nasolabial folds on both sides. This study attacked Arcface, CosFace, FaceNet, and VGGFace models. For the dodging attack on FaceNet in the physical world, the work could achieve an attack success rate of 100.0%. For the impersonation attack, the attack success rate is 55.32%. Finally, [43] also explored the effects of camera distance, sticker size, and head pose.

In summary, other papers use Gradient-based (e.g., L-BFGS [34], FGSM [36,37], FACESEC [42]), Visible Light-based [35,38,39], and GAN-based [8,40,41,43] methods. In the physical world, the model architecture and parameters are mandatory knowledge for the traditional Gradient-based method, which means it is only applicable to white-box attacks. This approach is not realistic for practical applications. The Visible Light-based method uses visible light projection to change the face feature pixels. Besides requiring many resources and projectors, this method is easily vulnerable to external environmental factors, such as an infrared cut-off lens leading to the attack’s failure. In contrast, we use a GAN-based approach to generate attack glasses or face patches, which is convenient. We also restrict the noise to the frame of the glasses (small area, not modified face features), which achieves a high success rate of attacks in both the physical and digital worlds. In addition, our method does not result in a “this person does not exist” warning in real-world face recognition systems. It is difficult for a layman such as a security guard to know the intent of our attack.

## 4. Proposed Method

The present study proposes a GAN-based attack method based on the generation of adversarial patches. The attack method assumes the use of a black-box model and is applicable to both the digital world and the physical world. It is shown that the proposed method achieves a success rate of 57.99% in digital dodging attacks and 48.78% in digital impersonation attacks. Moreover, the success rates for physical dodging and impersonation attacks are 81.77% and 63.85%, respectively. In other words, the attack performance of the proposed method is significantly better than that of previous methods reported in Section 3.

The present study refers to the model architecture used in [8,44], in which the training images comprise a face data set, and adversarial noise is added to each face using the conventional GAN method. In contrast to the method in [8], however, the generator in the present study generates adversarial noise only on glasses rather than on the entire face, and then adds these adversarial glasses to the face prior to judgement by the discriminator. The proposed method is thus more easily applied for attacks in the physical world.

**Figure 1:**
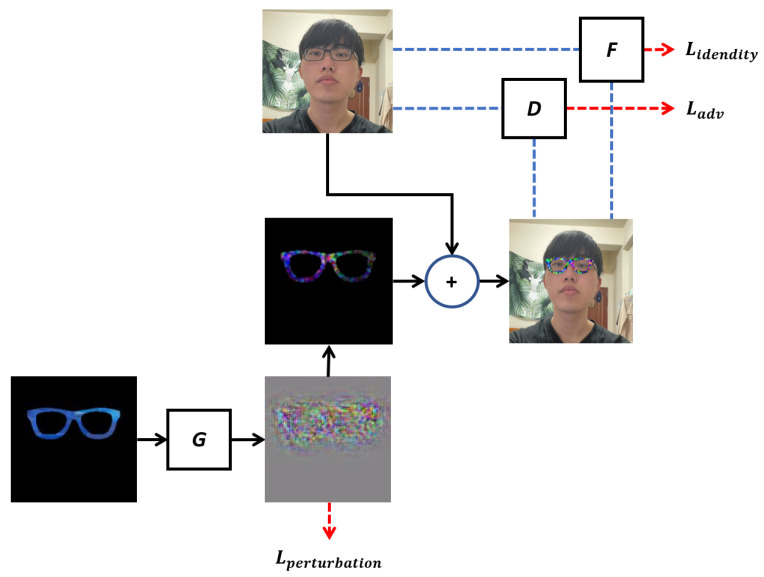
Proposed architecture.

On the whole, our proposed GAN architecture is illustrated in Figure 1, which comprises three main components: the generator *G*, the discriminator *D*, and the face matcher *F*. For the execution process of the proposed architecture, first, the glasses are the generator’s input for generating the perturbation. Second, the perturbation will be combined with the glasses and the person to form the merged image, which will be used as the input to the discriminator (*D*) and the face matcher (*F*). Third, the generator, discriminator, and face matcher will calculate the losses, $L_{perturbation}$, $L_{adv}$, and $L_{identity}$, respectively. The pseudo codes for generating adversarial glasses and patches are shown in Algorithms 1 and 2. The details of these components will be presented below.
**Algorithm 1** Training AdvFace in dodging attack**Input:**X Training Glasses Dataset*f* Training Face DatasetF Cosine similarity between an image pair obtained by face matcherG Generator with weight $G_{\theta }$D Discriminator with $D_{\theta }$*m* Batch sizeα Learning size  1:**for** number of training iterations **do**  2:   Sample a batch of probes ${\left\{x^{\left(i\right)}\right\}}_{i=1}^{m}∼X$  3:   Sample a batch of origin face images ${\left\{y^{\left(i\right)}\right\}}_{i=1}^{m}∼f$  4:   $\delta^{\left(i\right)}=G\left(x^{\left(i\right)}\right)$  5:   $x_{adv}^{\left(i\right)}=x^{\left(i\right)}+\delta^{\left(i\right)}$  6:   $L_{perturbation}=\frac{1}{m}[\sum_{i=1}^{m}max(P,||\delta^{\left(i\right)}{\left|\right|}_{2})]$  7:   $L_{identity}=\frac{1}{m}\left[\sum_{i=1}^{m}E\left[\left(F\left(y^{\left(i\right)},x_{adv}^{\left(i\right)}\right)\right)\right]\right]$  8:   $L^{D}=\frac{1}{m}\left[\sum_{i=1}^{m}\log\left(1-D\left(x_{adv}^{\left(i\right)}\right)\right)\right]$  9:   $L_{adv}=\frac{1}{m}\left[\sum_{i=1}^{m}\log\left(D\left(x^{\left(i\right)}\right)\right)+\log\left(1-D\left(x_{adv}^{\left(i\right)}\right)\right)\right]$10:   $L^{G}=L_{adv}+\lambda_{i}L_{identity}+\lambda_{p}L_{perturbation}$11:   $G_{\theta }=\mathrm{Adam}\left({▽}_{G}L^{G},G_{\theta },\beta_{1},\beta_{2}\right)$12:   $D_{\theta }=\mathrm{Adam}\left({▽}_{D}L^{D},G_{\theta },\beta_{1},\beta_{2}\right)$13:**end for**

**Algorithm 2** Training AdvFace in impersonation attack
**Input: **
X Training Face Dataset*f* Target Face DatasetF Cosine similarity between an image pair obtained by face matcherG Generator with weight $G_{\theta }$D Discriminator with $D_{\theta }$*m* Batch sizeα Learning size
  1:**for** number of training iterations **do**  2:   Sample a batch of probes ${\left\{x^{\left(i\right)}\right\}}_{i=1}^{m}∼X$  3:   Sample a batch of target images ${\left\{y^{\left(i\right)}\right\}}_{i=1}^{m}∼f$  4:   $\delta^{\left(i\right)}=G\left(x^{\left(i\right)},y^{\left(i\right)}\right)$  5:   $x_{adv}^{\left(i\right)}=x^{\left(i\right)}+\delta^{\left(i\right)}$  6:   $L_{perturbation}=\frac{1}{m}[\sum_{i=1}^{m}max(P,||\delta^{\left(i\right)}{\left|\right|}_{2})]$  7:   $L_{identity}=\frac{1}{m}\left[\sum_{i=1}^{m}E\left[1-F\left(y^{\left(i\right)},x_{adv}^{\left(i\right)}\right)\right]\right]$  8:   $L^{D}=\frac{1}{m}\left[\sum_{i=1}^{m}\log\left(1-D\left(x_{adv}^{\left(i\right)}\right)\right)\right]$  9:   $L_{adv}=\frac{1}{m}\left[\sum_{i=1}^{m}\log\left(D\left(y^{\left(i\right)}\right)\right)+\log\left(1-D\left(x_{adv}^{\left(i\right)}\right)\right)\right]$10:   $L^{G}=L_{adv}^{G}+\lambda_{i}L_{identity}+\lambda_{p}L_{perturbation}$11:   $G_{\theta }=\mathrm{Adam}\left({▽}_{G}L^{G},G_{\theta },\beta_{1},\beta_{2}\right)$12:   $D_{\theta }=\mathrm{Adam}\left({▽}_{D}L^{D},G_{\theta },\beta_{1},\beta_{2}\right)$13:
**end for**



### 4.1. Generator

The aim of the generator is to generate an adversarial image that causes the face recognition system to misclassify the input. A glasses image *x* is input to the generator, and the generator randomly generates G(x) from the multi-dimensional space. The noises generated by the generator are then added to the original glasses image to produce x+G(x). (That is, x+G(x) denotes that only noises within the glasses are remained on the glasses.) As shown in Figure 1, the generated noise, G(x), can be controlled by the $L_{perturbation}$ loss. In particular, the $L_{2}$ norm of G(x), designated as ${∥G\left(x\right)∥}_{2}$, is taken and compared with a predefined noise threshold, *P*. The loss function, $L_{perturbation}$, is then assigned based on the outcome of this comparison, as shown in the following:

(1) $$L_{perturbation}={E[max(P,∥G\left(x\right)∥}_{2})]$$

With the $L_{perturbation}$, the generator will be trained to generate as much noise as possible with the constraint that the $L_{2}$ norm of G(x) is close to but not larger than the predefined noise threshold, *P*.

The proposed architecture incorporates an additional loss, designated as $L_{identity}$, which aims to encourage the generator (*G*) to generate noise specifically intended to cause the face recognition system to misjudge the input face as that of another individual. The adversarial image *x + G(x)* is first attached to a face image *f* through image processing to produce *f + x + G(x)*. The matcher (*F*) then compares *f + x + G(x)* with the original face image *f* and calculates the difference between them as $L_{identity}$. The aim of the generator is to minimize the output value of the face matcher, *F*. The $L_{identity}$ here has different definitions for non-targeted attack and targeted attack. When generating adversarial glasses for a non-targeted attack, $L_{identity}$ is calculated as follows:

(2) $$L_{identity}=E[F\left(f,\left(f+x+G\left(x\right)\right)\right],$$

where *f* is the face image of the person performing adversarial attack.

However, when generating adversarial faces *((x+G(x), where $x=f_{t}$*) to carry out targeted attacks, the aim is to attack a specific target, and hence the input image patched with the adversarial face image, *f + x + G*(*x*), is compared to the face image of the attack target, $f_{t}$. Thus, in this case, $L_{identity}$ is computed as

(3) $$L_{identity}=E\left[1-F\left(f_{t},f+x+G\left(x\right)\right)\right].$$

For the layer structure of the generator, Table 3 shows the structural parameters of the design in detail.

### 4.2. Discriminator

The purpose of the discriminator is to compare the original face *f* and the face image with glasses *f + x + G* generated by the generator. The original face f is input to the discriminator, and the generator then attempts to minimize the difference between *f* and *f + x + G* such that the discriminator is unable to distinguish between them. In the present architecture, the discriminator is implemented using the loss function described in [9]. That is,

(4) $$L_{adv}=E\left[\log D(f)\right]+\left[\log(1-D(f+x+G(x)))\right]$$

For the layer structure of the discriminator, Table 4 shows the structural parameters of the design in detail.

### 4.3. Face Matcher

The propose of the face matcher is to quantify the similarity between two faces. In other words, *F* can be regarded as a form of face recognition system. To compare the similarity between two faces, the face matcher receives the two face images and outputs two corresponding feature vectors. The distance between the two feature vectors is then taken as a measure of the similarity between them, where a smaller distance indicates a greater similarity, and vice versa.

The total loss function of the proposed GAN architecture thus has the form

(5) $$L_{total}=L_{adv}+x_{1}L_{perturbation}+x_{2}L_{identity},$$

where $x_{1}$ and $x_{2}$ are weighting values used to control the relative contributions of the Lperturbation and Lidentity losses, respectively. The total loss, $L_{total}$, is then fed back to the generator *G* for further training. In particular, the generator produces a new adversarial image, which is reevaluated by the discriminator *D* and face matcher *F*. The resulting loss, $L_{total}$, is then returned to the generator as feedback once again. The training process continues iteratively in this way until the generator produces a set of high-quality adversarial images which are virtually indistinguishable from the original face images by both the discriminator and the face matcher.

## 5. Experimental Results

The present study constructed two system environments: one in the digital world and another in the physical world. The former system implemented a face recognition system for the digital world and a generator for producing adversarial patches. The system was implemented on the Ubuntu 18.04 operating system with 256 GB of memory space, a Tesla V100 GPU, 32 GB PCIe (NVIDIA Corp., San Jose, CA, USA), and an Dell PowerEdge R740 with Intel${}^{®}$ Xeon${}^{®}$ Silver 4116 CPU @ 2.10 GHz. The system was programmed in Python 3.6 using a variety of deep learning tools, including TensorFlow 1.14.0, Keras 2.3.1, Pytorch 1.9, and CUDA version 11.0.

The second system implemented a face recognition system in the physical world. For testing convenience, and to reproduce a realistic face recognition system environment, the system was implemented on an ASUS X556UR laptop (ASUSTek Computer Inc., Taipei, Taiwan) under an Anaconda virtual environment. The face recognition system was run on a NVIDIA GeForce 940MX (NVIDIA Corp., San Jose, CA, USA) 2 GB graphics card and was programmed in Python 3.6 with TensorFlow 1.14.0, Keras 2.3.1 and Pytorch 1.9. The laptop camera had a poor resolution of just 480 p. Thus, to enhance the face recognition process, the laptop was interfaced with a Logitech C925e (Logitech International S.A., Lausanne, Switzerland, and Newark, CA, USA) webcam with an improved resolution of 1080 p.

### 5.1. Evaluation Metric

The performance of the proposed GAN-based attack method was quantified by evaluating the attack success rate in both the digital world and the physical world. For both worlds, the attack success rate was investigated for both dodging attacks and impersonation attacks. In the case of dodging attacks in the digital world, the success rate was computed as follows:

(6) $$\frac{\sum_{i\in N}^{}\left({\hat{y}}_{i}\neq y_{i}\right)and\left(d\left({\hat{y}}_{i}\right)<threshold\right)}{\left|N\right|},$$

where $y_{i}$ is the original class of the input image *i*; ${\hat{y}}_{i}$ is the image class (of the image *i*) predicted by the face recognition model; $d({\hat{y}}_{i})$ is the similarity (e.g., cosine distance) of the input image *i* and an image in class ${\hat{y}}_{i}$; |N| is the total number of input images; and threshold is a threshold parameter used for classification judgement purposes. Note that the threshold parameter was assigned a value of 0.4, where a similarity less than this threshold was taken to indicate a valid classification result.

The success rate of impersonation attacks in the digital world was evaluated using Equation (7), in which $\tilde{y}$ is the class of the target, ${\hat{y}}_{i}$ is the class predicted by the face recognition model.

(7) $$\frac{\sum_{i\in N}^{}\left({\hat{y}}_{i}=\tilde{y}\right)and\left(d\left({\hat{y}}_{i}\right)<threshold\right)}{\left|N\right|}$$

In the physical world, the face images were read directly through the webcam. Thus, the adversarial patches produced by the generator were printed and worn by the subjects. For each subject, images were collected over a 10 s period with head motion allowed. For the dodging attacks, the attack was considered to be successful when the system identified three consecutive face images as belonging to an individual other than the subject. Similarly, for the impersonation attacks, the attack was considered to be a success when the system identified three consecutive face images as belonging to the target individual.

### 5.2. Datasets

Three datasets were used to construct the face recognition systems, namely one dataset consisting of 3000 face images chosen from the LFW open-source face dataset, and two self-collected small face datasets. The first self-collected database contained 10 subjects (6 male and 4 female) between the age of 22 and 27 years old, with five face images for each subject. The second self-collected database added an additional 12 individuals to the first dataset, giving a total of 22 individuals (16 male and 6 female) between the ages of 20 and 34 years old. Again, the dataset contained five face images for each individual. For both self-collected databases, the face images were captured in a well-lit indoor environment using a mobile phone camera.

When performing dodging attacks against the face recognition systems, adversarial patches were produced by adding noise to the glasses dataset in [34], which contains various styles of glasses, each with multiple colors, giving a total of 16,833 images. Meanwhile, the impersonation attacks were conducted using the 10 individuals in the first self-collected dataset as training subjects, where each individual wore printed adversarial glasses and face patches.

### 5.3. Face Recognition Systems in Digital World

**Figure 2:**
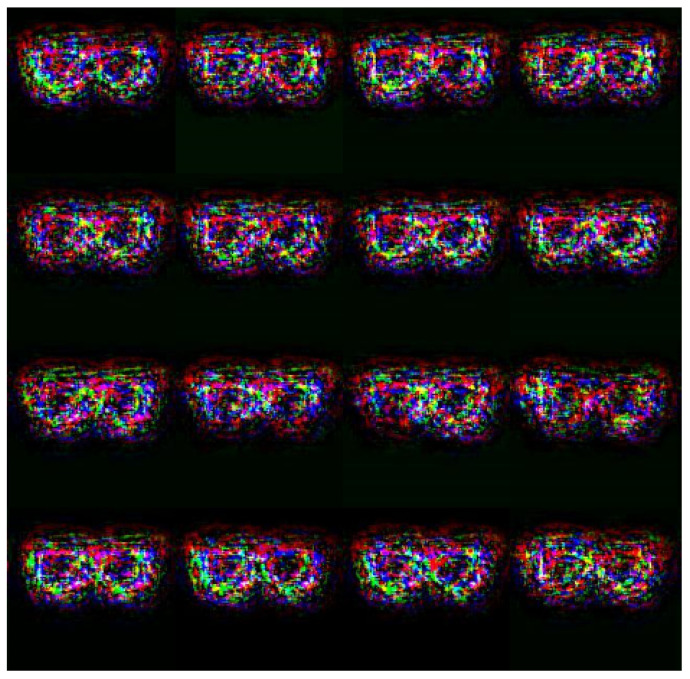
Generated adversarial glasses.

The experiments commenced by evaluating the attack performance of the proposed GAN-based adversarial patch method against the face recognition systems constructed in the digital world. The attack performance was evaluated for both the face recognition system built using the LFW dataset and the systems built using the two self-collected databases, respectively. In all three cases, face recognition was performed using the Deepface [45] open-source model, with a similarity (cosine distance) threshold set as 0.4 in order to achieve a False Accept Rate (FAR) of 0.1%. Two different adversarial patches were generated to carry out dodging attacks and impersonation attacks, respectively. The dodging attacks were conducted using the adversarial glasses patches shown in Figure 2.

**Figure 3:**
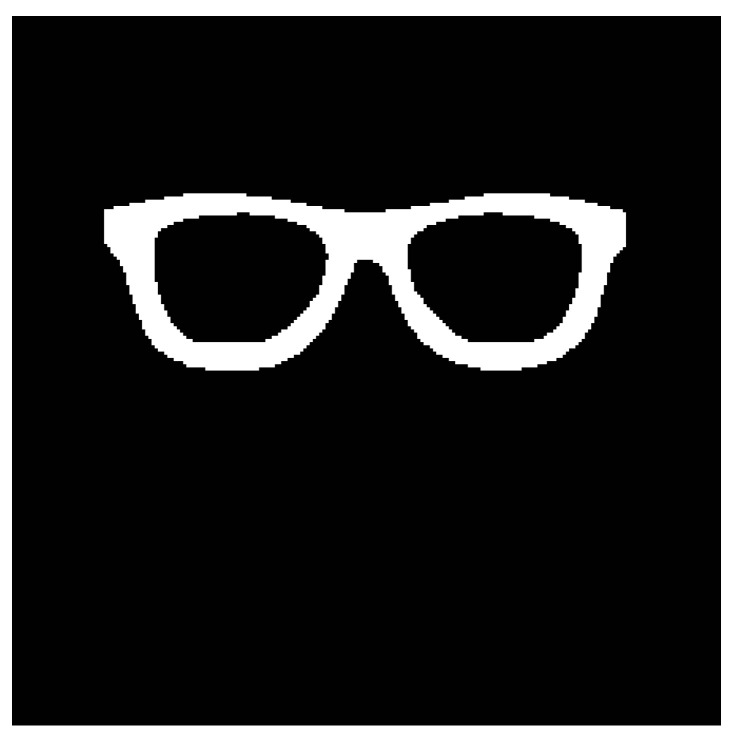
Glasses mask.

**Figure 4:**
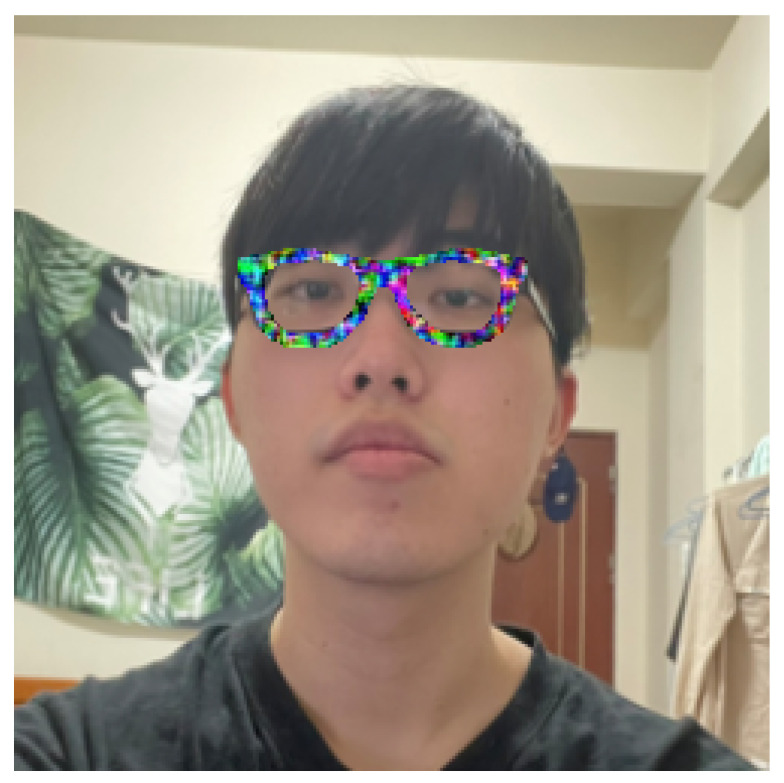
Addition of adversarial glasses in the digital world.

The adversarial glasses were passed through the mask shown in Figure 3, and the resulting frames were applied to the face images one-by-one to perform adversarial attacks, as shown in Figure 4.

**Figure 5:**
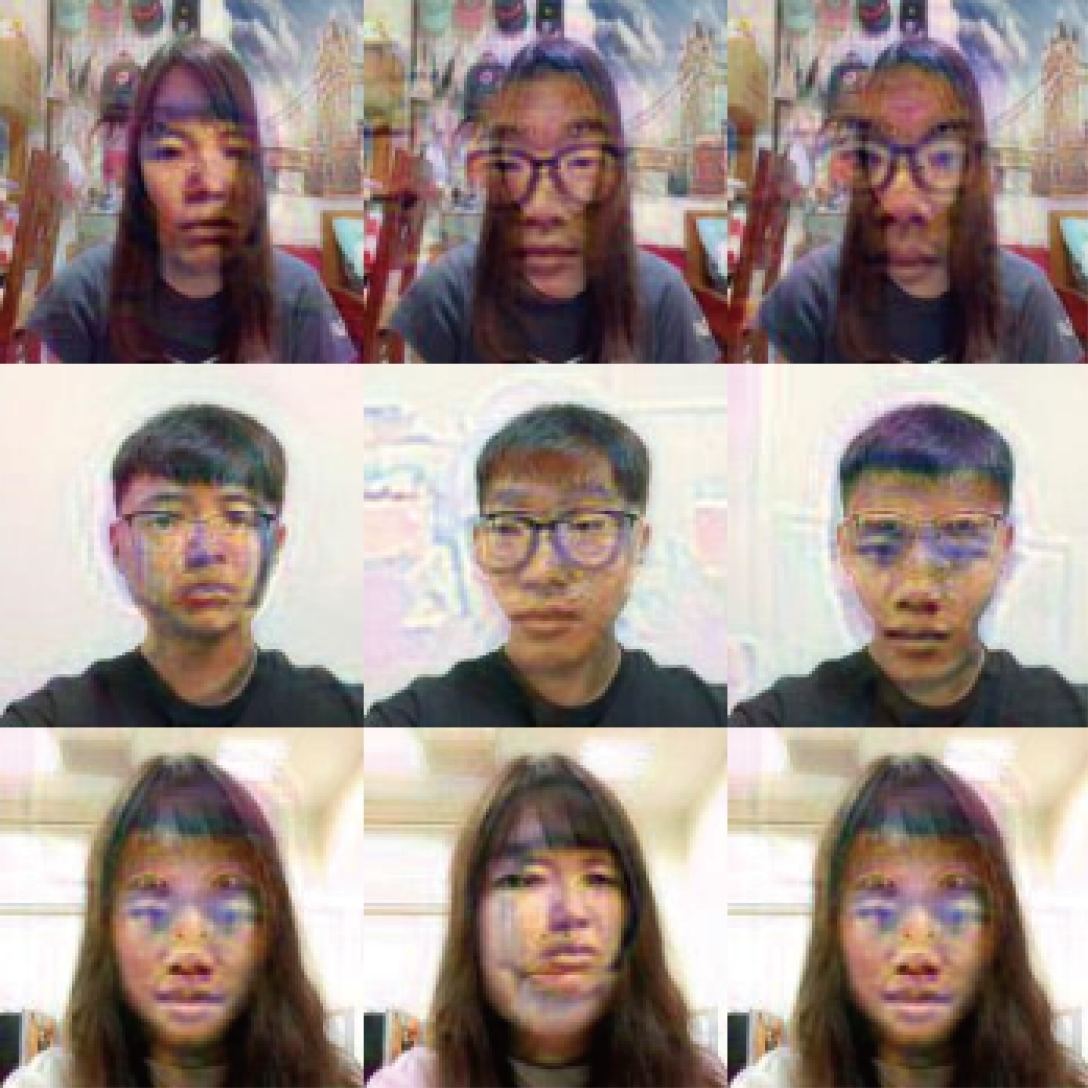
Generated adversarial faces.

**Figure 6:**
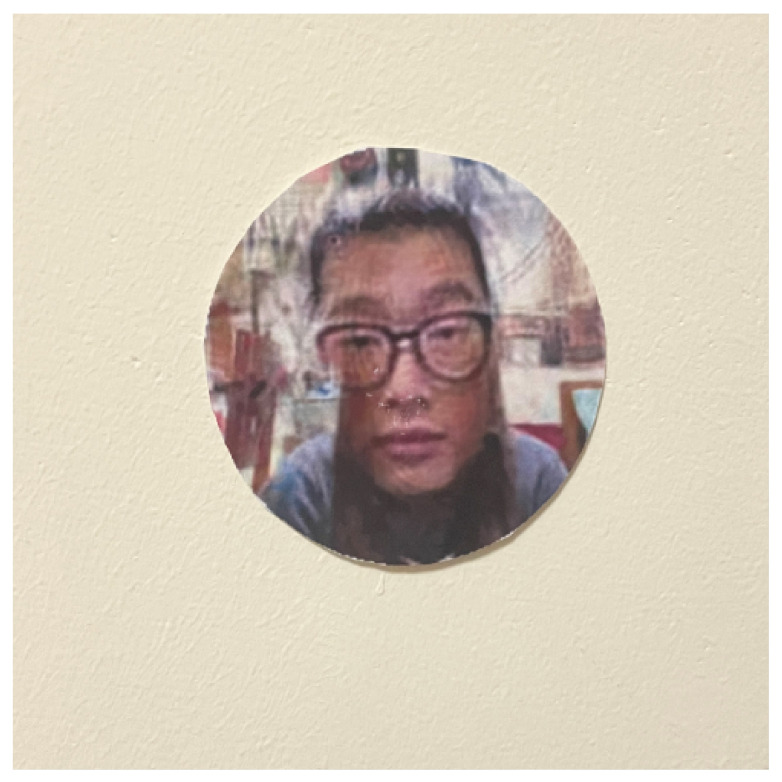
Adversarial faces cut into circles.

**Figure 7:**
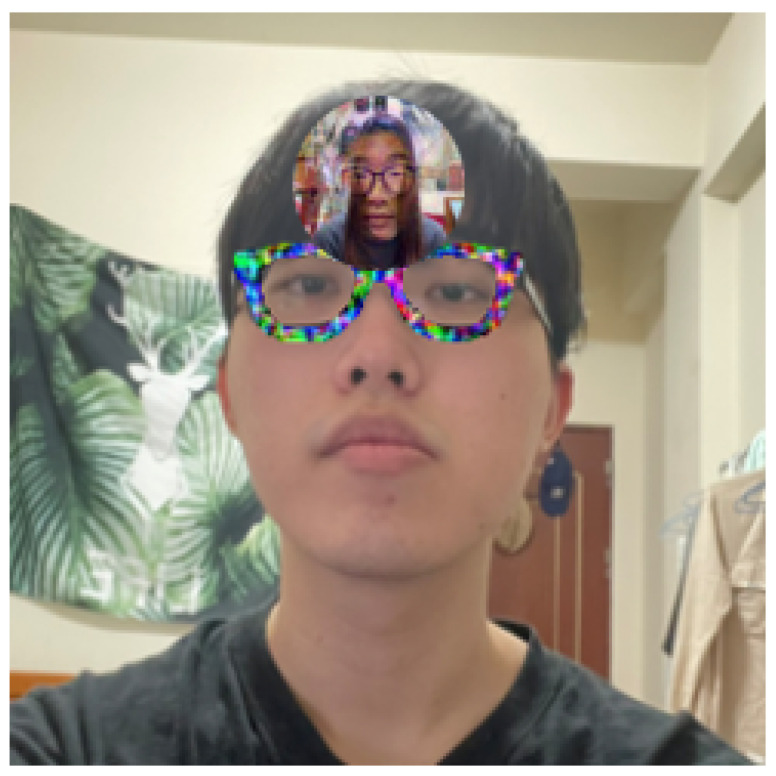
Addition of adversarial faces to face images with adversarial glasses in the digital world.

To carry out the impersonation attacks, adversarial faces were generated by the proposed method, as shown in Figure 5. The adversarial faces were extracted as circles (see Figure 6) and were then attached to the forehead regions of the face images with adversarial glasses, as shown in Figure 7.

### 5.4. Face Recognition Systems in Physical World

**Figure 8:**
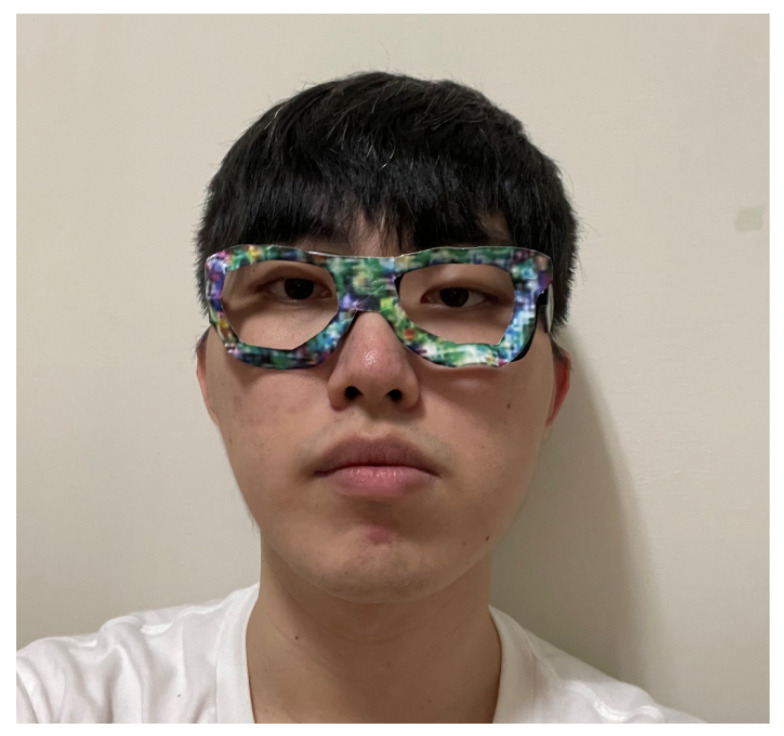
Wearing of adversarial glasses in the physical world.

**Figure 9:**
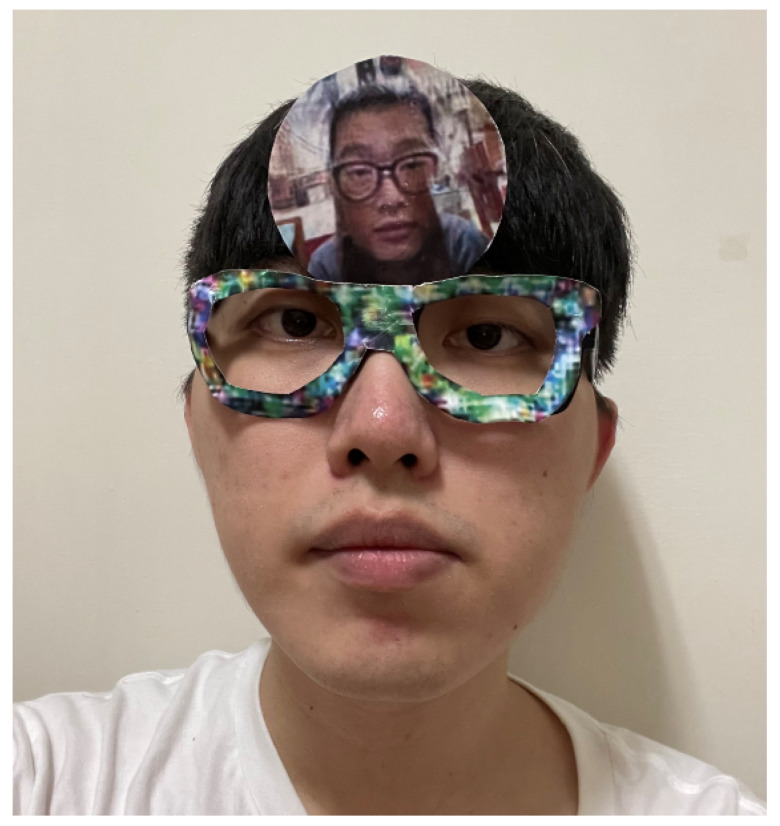
Wearing of adversarial glasses and an adversarial face in the physical world.

To evaluate the performance of the proposed model in the physical world, the face recognition system was constructed using the self-collected dataset containing 22 individuals. In implementing the face recognition system, the similarity threshold was set as 0.1 to achieve a FAR of 0.1%. For the dodging attacks, the adversarial glasses were printed on cardboard and worn around the eyes, as shown in Figure 8. For the impersonation attacks, the adversarial faces were additionally printed and attached to the forehead region, as shown in Figure 9.

### 5.5. Results

#### 5.5.1. Dodging Attacks in Digital World

The dodging attacks in the digital world were conducted against all three face recognition systems based on the LFW dataset (3000 images), Self-Collected Dataset 1 (10 subjects), and Self-Collected Dataset 2 (22 subjects), respectively. The intention of using the three different face recognition systems was to evaluate the relationship between the number of faces in the face recognition database and the attack success rate. The experiments commenced by evaluating the attack performance against the LBW face recognition system. The attack was conducted using 10 face images chosen at random from the LBW dataset, where each image wore 64 different adversarial glasses in turn. The corresponding attack results are shown in Table 5.

Overall, the results presented in Table 5 show that the adversarial glasses result in a high attack success rate for some individuals (e.g., #1, #9 and #10), but a low attack success rate for others (e.g., #2 and #4). A detailed analysis revealed two main reasons for the low success rate in these cases: (1) even after wearing adversarial glasses, the adversarial face was still very similar to the original; and (2) the LFW dataset contained no faces similar to the adversarial face with glasses, i.e., the dataset contained no target to attack. Accordingly, the dodging attacks were repeated against the face recognition systems built using the two self-collected datasets, respectively. The corresponding results are presented in Table 6 and Table 7.

As shown, the average attack success rates against the 10-person and 22-person databases are 24.18% and 57.99%, respectively. In general, in conducting successful dodging attacks, the aim is for the face recognition system not only to identify the original face image after the addition of adversarial glasses, but also to match the face with another face. For the face recognition system constructed with a larger number of faces, there exist more targets which can be matched by the adversarial face. Consequently, as the size of the database used by the face recognition system increases, the vulnerability of the system to adversarial glasses attacks also increases.

#### 5.5.2. Impersonation Attacks in Digital World

The impersonation attacks were conducted against the face recognition system built using the self-collected database of 22 individuals. Ten individuals were chosen randomly from the database for testing purposes. In addition to wearing adversarial glasses, each face also wore the adversarial faces of the other nine individuals in turn. That is, each individual attacked nine targets. The corresponding attack results are shown in Table 8.

**Figure 10:**
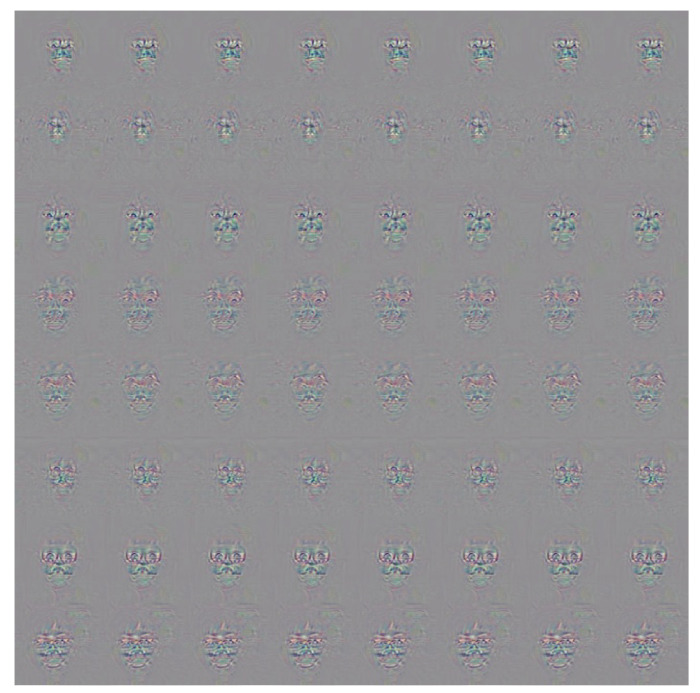
Generated adversarial masks.

It is seen in Table 8 that the overall average success rate is 48.78%. However, it is also noted that some of the targets (e.g., #3 and #8) are less easily impersonated than others. It is speculated that when adversarial face stickers are generated by the method of this study, some faces are more difficult to attack. Furthermore, some of the adversarial images also have a lower attack success rate than others. For example, the successful attack rate of adversarial image #6 is just 23.3%. In other words, the face recognition system matches the adversarial face image with the original image rather than the target individual. To investigate this phenomenon further, the method proposed in [8] was used to generate adversarial noise for all of the faces in the self-collected database, as shown in Figure 10.

It can be seen that some of the faces are attacked by generating noise in the eye region of the image, while in other cases, the noise is distributed over the entire face. However, the method proposed in the present study generates adversarial glasses and stickers which are applied only to certain regions of the face, and it cannot attack those faces with the noise is distributed over the entire face.

#### 5.5.3. Dodging Attacks in Physical World

The dodging attack success rate in the physical world was evaluated for both self-collected databases. For each database, 10 subjects were selected for testing purposes, where each subject wore 11 adversarial glasses in turn. The corresponding attack success rates are shown in Table 9 and Table 10, respectively.

In the attacks performed in the physical world, the subjects were allowed to turn their face during the detection process. Thus, compared to the digital case, in which the detection process was limited to only a single face input image, the detection process in the physical world was less constrained. As shown in Table 9 and Table 10, the average dodging attack success rates against the 10-person database and 22-person database were 39.94% and 81.77%, respectively. In other words, as for the attacks performed in the digital world, the attack success rate in the physical world also increases as the size of the database increases. The results also imply that when the subject turns the face to a non-frontal angle, the likelihood of the face recognition system misclassifying the input face image also increases.

#### 5.5.4. Impersonation Attacks in Physical World

The performance of the impersonation attacks in the physical world was evaluated using the 10 subjects in the first self-collected database as test subjects and the 22 individuals in the second self-collected database as targets. Each of the test subjects wore adversarial glasses and the adversarial faces of all the other subjects in the first self-collected database in turn. The aim of the attack was to cause the face recognition system to recognize the target on the adversarial face sticker rather than the original subject. The corresponding attack results are presented in Table 11.

As shown, the average success rate of the impersonation attacks is 63.85%. Interestingly, the results show that even though the same adversarial face patch of a given target is added to all of the test subjects, the attack success rates are different for different test subjects. On the other hand, it is also evident that some subjects (e.g., #5, #6, #7) are easier to attack than others (e.g., #3). Notably, we achieve more than 70% attack success rate in the physical world on most of the targets, even if we set a threshold of 0.4, corresponding to a False Accept Rate (FAR) of 0.01%. Moreover, since the above table is a comprehensive attack success rate test for each person against others, the existing literature mainly discusses the attack success rate of a single target. Therefore, for a more intuitive comparison with the existing literature, we show in Table 12 that the average attack success result for a single target is 78%.

### 5.6. Comparison of Dodging Attack Success Rates of Different Methods

Table 13 compares the dodging attack success rate of the proposed method in the physical world with that of several other attack methods proposed in the literature.

The method proposed in [34] applied adversarial glasses to the test images and achieved an average attack success rate of 97.22%. However, the adversary attack was a white-box attack, which is unrealistic in practical attack environments. The premise of the white-box attack is to know the model architecture and parameters, which is worlds apart from our black-box attack. At the same time, through the experiments of cosine similarity in the [36], we can observe some interesting variations of cosine similarity. In [36], cosine similarity varies between white box and black box attacks (note that when the cosine similarity is larger, the more similar it is). First, experiments in [36] are based on semi-white-box attacks, and we can see that the cosine similarity ranges from 0.15 to 0.2 in the Final similarity. Second, when the authors transfer the adversarial attack noise generated on the white-box attack to other unknown models, the architecture and parameters of the unknown model are unknown for the adversarial attack noise. In this case, it can be considered a no-box attack (more rigorous black box attack), which yields a cosine similarity of about 0.4. However, the no-box’s cosine similarity increases from 0.2 to 0.4, which means that the similarity between the original image and the adversarial image is affected. That is, the attack success rate may be slightly decreased. In other words, the reason why our proposed attack success rate is lower than [34] is due to the difference between white-box and black-box. Moreover, the performance evaluation was conducted using just three test subjects in [34], which had slightly fewer test subjects. The methods in [38,39] adopted a black-box attack model and considered a greater number of test subjects (9 and 10 subjects, respectively). They were thus more representative of real-world attack scenarios than the method in [34]. Moreover, they achieved reasonable attack success rates of 85.7% and 70%, respectively. However, both methods require the use of visible light projection systems when conducting the attack and need careful consideration of the face angle and mask conditions. Thus, neither method is practical in real-world physical attack situations. In addition, we further compared our work with [38]. The work [38] did not set a similarity threshold. As aforementioned, the attack success rate will be higher without a similarity threshold. However, it is not practical as real-world face recognition systems will set the threshold properly. Therefore, we conducted an experiment by varying the threshold from 0 to 1 with an interval of 0.05. The purpose of this experiment is to illustrate the relationship between attack success rate and threshold. We discuss the results in Section 5.7. Tong et al. [42] proposed the FACESEC method based on gradient $l_{0}$-norm for adversarial patch attacks. However, this type of attack is significantly affected by the adversarial patch’s wearing position, shape, and scale. Although in [42], whose attack method (patch) and position (eyeglass frame) are the same as ours, the attack success rate in [42] is lower than ours, at only 54%. The reason is the difference between Gradient-based and GAN-based. More interestingly, for the advantages and disadvantages of Gradient-based and GAN-based approaches, it has been shown in the experimental results of [40] that the performance of gradient-based attacks will be slightly lower than that of GAN-based approaches. Clearly, our results are consistent with the results of [42]. By contrast, the method proposed in this study not only considers a black-box attack model and achieves a relatively high success rate of 81.77% over 10 test subjects, but also requires only the use of simple temporary adversarial glasses stickers to deceive the face recognition system. It is thus more convenient and practical than the other methods presented in Table 13, while retaining a similar (if not better) attack performance.

Table 14 compares the impersonation attack success rate of the proposed method in the physical world with that of four other attack methods reported in the literature.

The method in [34] used a white-box attack model to generate adversarial glasses and achieved an average attack success rate of 75%. It is noted that the attack rate is slightly higher than that of the present study (63.85%). However, the present study is based on the more realistic assumption of a black-box model and, moreover, considers a greater number of test subjects (10 vs. 3 subjects), with the consequence that the results are expected to be more reliable. Although the average attack success rate of the combined experimental results is slightly lower than that of [34], the reason lay in the fact that we performed a comprehensive attack test on several targets and averaged the resulting attack success rates. For the above reasons, we compared the attack success rate of the single target with [34], as shown in Table 14, which resulted in 78% and had better results than that of Sharif et al. [34]. The method in [38] also considered a black-box attack model and used visible light to produce noise. Moreover, the attack evaluation considered a relatively large number of test subjects (9 subjects). However, the attack success rate against the FaceNet recognition system was just 32.4%, i.e., around half that of the present study. The authors in [39] also used projected light to produce noise in order to deceive the face recognition system. The attack success rate was 60%, and is thus close to that obtained in the present study. However, the evaluation process in [39] considered only 1 subject tested 10 times, and hence the evaluation results may not be reliable. Furthermore, as for the method in [39], the attack requires the use of visible light projection equipment, which is impractical for most real-world situations. By contrast, the present method requires only the use of adversarial stickers (glasses and face), which can be easily removed once the face recognition system has been fooled. The method in [40] requires only the application of makeup to the face, and is thus also convenient in real-world attack scenarios. However, the attacks in [40] were performed only once for seven different face angles, and hence the reliability of the evaluation results cannot be guaranteed. Moreover, the attack success rate was just 52.82%, and is hence lower than that of the present study (63.85%). Finally, in [43], the GAN-based adversarial stickers were crafted and put on five regions near to the facial organs (i.e., two superciliary arches, two nasolabial sulcus, and the nasal bone). Notably, these regions are critical regions for face recognition [8,33]. As a result, [43] was able to achieve 100% attack success rate of the dodging attack in the physical world which are higher than our results. However, the success rate for the impersonation attack is only 55.32%, which is slightly lower than ours.

#### Defense Mechanism

The attack method proposed in the present study exploits adversarial patches, which occupy only small regions of the face, rather than adversarial examples, which occupy the entire face. Many defense mechanisms based on face recognition rely on the detection of live subjects through temperature measurements [46], or the detection of adversarial samples [47]. These methods thus have only a limited ability to counter the adversarial patch-based method proposed in the present study. Accordingly, this study also proposes a new defense method, in which it is assumed that the defender already knows the attack method employed by the adversary. A new class, referred to as “Defense”, is added to the output label. In particular, photos of each subject wearing adversarial glasses are added to the face database to counter dodging attacks, while photos of each subject wearing adversarial glasses and adversarial faces are also added to the face database to thwart impersonation attacks. All these inputs are labeled as “Defense” during training of the face recognition system. Table 15 and Table 16 show the dodging attack success rate and dodging attack defense rate, respectively, following the implementation of the proposed defense mechanism.

The results presented in Table 15 show that the dodging attack success rate reduces significantly from 57.99% to 0.06% following the implementation of the proposed defense mechanism. Moreover, the average defense rate is 89.69%. In other words, the proposed defense mechanism significantly improves the robustness of the face recognition system against dodging attacks.

Table 17 and Table 18 show the equivalent results for impersonation attacks.

As shown, the implementation of the defense mechanism reduces the average impersonation attack success rate from 48.33% to 28.33% and achieves an average defense rate of 41.1%. It is noted that the defense rate is lower than that for dodging attacks. Nonetheless, the defense mechanism still reduces the original attack success rate by around 60%.

It can be shown that, the attack success rate attack success rate dropped from 48.33% to 28.33%, and the defense rate is 41.1%, the defense is not as effective as dodging attack, but it still can reduce the original attack by about 60%, and defense about 40% attack.

### 5.7. Threshold

In our study, we additionally discuss two issues, which are (1) the relationship between threshold and attack success rate and (2) the portability of attacking other models by no-box.

**Figure 11:**
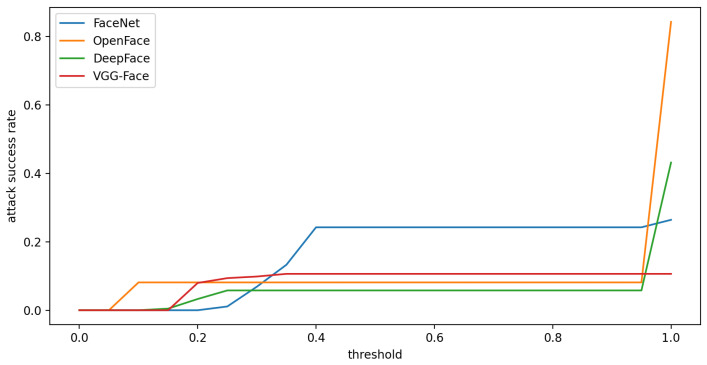
Compare the relationship between threshold and attack success rate.

First, about the relationship between threshold and attack success rate, we can observe from Figure 11 that the threshold directly affects the attack success rate. When the threshold is set smaller, the attack success rate is lower. Based on the observation, setting a proper threshold is necessary for the face recognition system. This also shows that our attack method is more realistic than [38] by properly setting the threshold.

Second, according to the definition of [48], a no-box attack does not query the face recognition system. That is, when generating adversarial samples, it does not refer to the confidence scores of the target face recognition system. Therefore, when our adversarial glasses attack other face recognition systems, that forms a no-box attack. Based on the experimental results shown in Figure 11, we can observe that our attack method exhibits transferability.

### 5.8. Time Efficiency

For the analysis of techniques, other papers adopted Gradient-based (e.g., L-BFGS [34,42]), Visible-light-based [38,39], and GAN-based [40,43] methods. For Gradient-based, [34] took 4.39 h to output 35 attack images. The paper [42] did not mention its time efficiency. For the Visible Light-based approach, the paper [38] showed that VLA took less than 3 s on average to generate a frame pair containing a perturbation frame and a concealing frame. However, the paper [39] did not describe its time efficiency. For GAN-based, papers [40] neither discussed their time efficiency. [43] took 26 min to generate 8100 stickers. For our approach, first, one iteration could generate 64 pairs of glasses, which took 75 s. In all experiments, we ran 50 iterations. The total running time is 62.5 min. Second, for adversarial patches, each iteration could generate 32 adversarial patches in 20 s. It took 66.67 min to run 200 iterations. For the GAN model (including the Facenet) we used, the number of parameters is 31529204, and the FLOPs reach 58332210.

## 6. Conclusions

This work proposes an attack method based on GAN that generates noise and restricts the adversarial glasses and patches generated in the face region. This method can achieve black-box attacks in both digital and physical worlds. Among the attacks in the physical world, our method is more representative of the real-world attacks in the physical world by wearing adversarial glasses or patches on the face during face recognition. Furthermore, due to the assumption of a black-box model, the adversary requires no knowledge of the parameters and structure of the deep learning model employed by the face recognition system to conduct an attack.

On the other hand, the reason why the traditional method of dodging attacks fails is that “this person does not exist” attracts the attention of the guards, which is a severe problem. However, in dodging attacks, our method can make the face recognition system mistakenly identify the attacker as someone else, thus posing a significant threat to the face recognition system. Moreover, we take advantage of the fact that people often use glasses as a fashion accessory to hide the adversarial perturbation in the glasses. As such, it is difficult for laypersons to know our attack intentions. In impersonation attacks, we can wear glasses and patches that disguise a person as other people without tampering with focused features on the face. Namely, we can be who we want to be in front of the face recognition system. Furthermore, face recognition is used in various fields nowadays. Through our experiments, we have verified that when the database of the face recognition system is larger, the chances of being hacked will increase. In this case, serious security concerns still need to be considered and improvements need to be made.

In another exploration, we introduce a defense mechanism to counter the GAN-based adversarial patch method. The results show that the proposed mechanism detects almost all dodging attacks and more than half of the impersonation attacks. In impersonation attacks, although the adversarial patches applied in this study occupy only a small region of the face, which is still easily recognized by the supervisor monitoring the face recognition system. In this case, the attack is easy to fail. Therefore, future works will generate less obvious adversarial patches to improve the attack’s success rate in the supervisor’s presence.

## Figures and Tables

**Table 1:** Previous works of face recognition.

Previous Works	Database	Method	Accuracy	Year
[10]	Tufts face	MvRDTSVM	91.55%	2022
		MvFRDTSVM	88.82 %	2022
[11]	AT&T face	DWT + PCA + SVM	96%	2018
		DWT + LDA + SVM	96%	2018
		DWT + ICA + SVM	94.5%	2018
[12]	PubFig83	CSV-DML	84.6%	2022
[13]	LFW	DeepFace-ensemble	97.35%	2014
[14]	LFW	Siamese Network (ZFNet + Inception-v1)	99.63%	2015
[15]	VGGFace2	ResNet-50	99.6%	2018
[16]	LFW	LightCNN-v29	98.98%	2020
[17]	VGGFace2-FP	PDA	95.32%	2020
[18]	VGGFace2-FP	HOG + Autoencoders	99.60%	2017
[19]	CASIA NIR-VIS2.0	CpGAN	96.63%	2020
[20]	LFW	FI-GAN	99.6%	2020
[21]	IJB-A	DAC	0.976 ± 0.01%	2020
[22]	YTF	ADRL	96.52 ± 0.54%	2017

**Table 2:** Related Works.

Related Works	Attack Method	Situation	Generate Object	Adversarial Capacity
[34]	L-BFGS	Physical	Patches	White-Box
[35]	LED	Physical	LED Infrared	White-Box
[36]	FGSM	Physical	Patches	White-Box
[37]	MI-FGSM	Physical	Patches	White-Box
[38]	VLA	Physical	Visible Light	Black-Box
[39]	Transformation-Invariant Adversarial Pattern	Physical	Visible Light	Black-Box
[8]	GAN-based	Digital	Digital Face Image	Black-Box
[40]	GAN-based	Physical	Adv-Makeup	Black-Box
[41]	GAN-based	Digital	Digital Image	Black-Box
[42]	FACESEC	Physical	Eyeglass	Black-box
[43]	GAN-based	Physical	Sticker	Black-box

**Table 3:** Structural parameters of the generator.

Layer	Type	Filters/Neurons	Stride	Padding
1	Conv	64 (kernel size = 7)	1	3
2	Conv	128 (kernel size = 4)	2	-
3	Conv	256 (kernel size = 4)	3	-
-	residual block	kernel size = 3	-	-
-	residual block	kernel size = 3	-	-
-	residual block	kernel size = 3	-	-
4	Unsampling	128 (kernel size = 5)	1	2
5	Unsampling	64 (kernel size = 5)	1	2
6	Conv	3 (kernel size = 7)	1	3

**Table 4:** Structural parameters of the discriminator.

Layer	Type	Filters/Neurons	Stride	Padding
1	Conv	32 (kernel size = 4)	2	-
2	Conv	64 (kernel size = 4)	2	-
3	Conv	128 (kernel size = 4)	2	-
4	Conv	256 (kernel size = 4)	2	-
5	Conv	512 (kernel size = 4)	2	-
6	Conv	1 (kernel size = 1)	1	-

**Table 5:** Digital dodging attacks against LFW database.

Number	Attack Success Rate
No.1	92.18%
No.2	1.56%
No.3	10.93%
No.4	4.68%
No.5	35.93%
No.6	32.81%
No.7	62.5%
No.8	85.93%
No.9	100%
No.10	93.75%
Average	52.02%

**Table 6:** Digital Dodging Attack in 10 people’s database.

Number	Attack Success Rate
No.1	0%
No.2	31.25%
No.3	78.13%
No.4	0%
No.5	37.5%
No.6	0%
No.7	0%
No.8	28.13%
No.9	34%
No.10	32.81%
Average	24.18%

**Table 7:** Digital Dodging Attack in 22 people’s database.

Number	Attack Success Rate	Number	Attack Success Rate
No.1	10.9%	No.12	0%
No.2	100%	No.13	98.4%
No.3	100%	No.14	1.5%
No.4	43.75%	No.15	100%
No.5	57.8%	No.16	100%
No.6	0%	No.17	100%
No.7	1.5%	No.18	96.8%
No.8	100%	No.19	39%
No.9	56.2%	No.20	95.3%
No.10	54.6%	No.21	34.3%
No.11	35.9%	No.22	50%
Average		57.99%	

**Table 8:** Digital impersonation attacks against 22-person face recognition system.

	**Attack Number**											
**Origin Number**		No.1	No.2	No.3	No.4	No.5	No.6	No.7	No.8	No.9	No.10	Average
	No.1		90%	10%	30%	70%	90%	60%	0%	30%	70%	50%
	No.2	50%		10%	30%	60%	90%	60%	30%	50%	60%	50%
	No.3	30%	80%		10%	60%	80%	60%	10%	60%	50%	48.9%
	No.4	70%	90%	30%		80%	90%	60%	10%	30%	70%	58.9%
	No.5	40%	70%	10%	30%		80%	60%	20%	40%	60%	45.6%
	No.6	10%	50%	0%	20%	40%		40%	30%	10%	10%	23.3%
	No.7	50%	80%	20%	30%	50%	70%		0%	10%	50%	40%
	No.8	10%	90%	10%	20%	60%	90%	50%		30%	70%	47.8%
	No.9	70%	90%	10%	40%	40%	80%	40%	10%		80%	51.1%
	No.10	30%	100%	20%	30%	70%	90%	60%	20%	50%		52.2%
											Total	48.78%

**Table 9:** Physical dodging attacks against 10-person face recognition system.

Number	Attack Success Rate
No.1	45.4%
No.2	54.5%
No.3	36.3%
No.4	27.2%
No.5	36.3%
No.6	54.5%
No.7	18.1%
No.8	45.4%
No.9	27.2%
No.10	54.5%
Average	39.94%

**Table 10:** Physical dodging attacks against 22-person face recognition system.

**Number**	**Attack Success Rate**
No.1	81.8%
No.2	72.7%
No.3	63.6%
No.4	100%
No.5	72.7%
No.6	81.8%
No.7	81.8%
No.8	90.9%
No.9	81.8%
No.10	90.9%
Average	81.77%

**Table 11:** The attack success rate of a single target for impersonation attacks in the physical world on a face recognition system with 22 persons in the database.

	**Attack Number**											
**Origin Number**		No.1	No.2	No.3	No.4	No.5	No.6	No.7	No.8	No.9	No.10	Average
	No.1		90%	0%	50%	90%	90%	80%	40%	90%	30%	62.2%
	No.2	50%		0%	40%	80%	90%	60%	50%	90%	90%	63.3%
	No.3	70%	80%		50%	90%	90%	90%	40%	90%	30%	70%
	No.4	60%	100%	20%		100%	90%	70%	40%	100%	80%	73.3%
	No.5	40%	80%	20%	40%		80%	90%	40%	90%	20%	55.5%
	No.6	40%	80%	10%	50%	80%		80%	40%	90%	20%	54.4%
	No.7	50%	90%	30%	50%	90%	90%		30%	90%	50%	63.3%
	No.8	40%	70%	10%	50%	90%	90%	90%		80%	60%	64.4%
	No.9	20%	100%	40%	50%	90%	90%	80%	40%		70%	64.4%
	No.10	40%	100%	30%	50%	80%	90%	90%	40%	90%		67.7%
											Total	63.85%

**Table 12:** In 22-person face recognition system, the attack success rate of a single target for impersonation attacks in the physical world.

Original No.	Attack Target	Attack Success Rate
No.1	No.3	70%
No.2	No.10	100%
No.3	No.9	40%
No.4	No.6	50%
No.5	No.4	100%
No.6	No.7	90%
No.7	No.5	90%
No.8	No.2	50%
No.9	No.4	100%
No.10	No.2	90%
Average		78%

**Table 13:** Comparison of physical dodging attack success rates of different methods.

Literature	Generate Object	Face Recognition Model	Adversarial Capacity	Number of Subjects	Number of People in Database	Dodging Attack’s Success Rate
[34]	Patches	VGG-Face	White-Box	3	10	97.22%
[38]	Visible Light	FaceNet	Black-Box	9	5749 (LFW)	85.7%
[39]	Visible Light	Commercial	Black-Box	10	50	70%
[42]	Eyeglass	FaceNet	Black-Box	10	5749 (LFW)	54%
Ours	Patches	FaceNet	Black-Box	10	22	81.77%

**Table 14:** Comparison of physical impersonation attack success rates of different methods.

Literature	Generate Object	Face Recognition Model	Attack Subjects	Number of Subjects	Number of Attack Target	Number of People in Database	Impersonation Attack’s Success Rate
[34]	Patches	VGG-Face	White-Box	3	1	10	75%
[38]	Visible Light	FaceNet	Black-Box	9	60	5749 (LFW)	32.4%
[39]	Visible Light	Commercial	Black-Box	25	1	50	60%
[40]	Adv-Makeup	Commercial	Black-Box	1	1	20	52.92%
[43]	Sticker	FaceNet	Black-Box	20	3	20 (VolFace)	55.32%
Ours	Patches	FaceNet	Black-Box	10	10	22	63.85%
				10	1	22	78%

**Table 15:** Dodging attack success rate after defense.

Number	Attack Success Rate
No.1	0%
No.2	0%
No.3	6.25%
No.4	0%
No.5	0%
No.6	0%
No.7	0%
No.8	0%
No.9	0%
No.10	0%
Average	0.06%

**Table 16:** Dodging defense rate after defense.

Number	Defense Rate
No.1	100%
No.2	81.25%
No.3	85.93%
No.4	78.12%
No.5	100%
No.6	56.25%
No.7	100%
No.8	100%
No.9	100%
No.10	95.3%
Average	89.69%

**Table 17:** Impersonation attack success rate after defense.

	**Attack Number**											
**Origin Number**		No.1	No.2	No.3	No.4	No.5	No.6	No.7	No.8	No.9	No.10	Average
	No.1		50%	10%	30%	0%	80%	30%	0%	0%	70%	30%
	No.2	50%		0%	30%	10%	90%	10%	0%	0%	60%	27.7%
	No.3	20%	80%		10%	0%	80%	20%	0%	0%	80%	32.2%
	No.4	50%	70%	20%		10%	90%	20%	0%	0%	70%	37.8%
	No.5	30%	50%	10%	30%		80%	10%	0%	0%	60%	30%
	No.6	10%	30%	0%	20%	0%		10%	0%	0%	10%	8.9%
	No.7	10%	50%	20%	30%	0%	70%		0%	0%	50%	25.6%
	No.8	10%	70%	10%	10%	0%	90%	0%		0%	70%	28.9%
	No.9	10%	70%	0%	40%	0%	80%	0%	0%		80%	31.1%
	No.10	30%	70%	20%	30%	0%	90%	30%	10%	0%		31.1%
											Total	28.33%

**Table 18:** Impersonation defense rate after defense.

	**Attack Number**											
**Origin Number**		No.1	No.2	No.3	No.4	No.5	No.6	No.7	No.8	No.9	No.10	Average
	No.1		40%	0%	0%	70%	10%	30%	40%	80%	0%	34.4%
	No.2	30%		10%	20%	80%	0%	90%	90%	10%	10%	37.8%
	No.3	10%	10%		0%	60%	0%	50%	90%	90%	0%	33.3%
	No.4	20%	20%	10%		80%	10%	70%	90%	10%	0%	34.4%
	No.5	60%	50%	60%	20%		20%	80%	80%	10%	20%	44.4%
	No.6	90%	70%	10%	70%	90%		90%	10%	10%	90%	58.9%
	No.7	90%	50%	70%	30%	90%	30%		90%	10%	40%	55.6%
	No.8	0%	20%	10%	0%	60%	0%	50%		80%	0%	24.4%
	No.9	80%	30%	70%	20%	90%	20%	10%	80%		20%	46.7%
	No.10	40%	30%	40%	20%	80%	0%	60%	90%	10%		41.1%
											Total	41.1%

## Data Availability

Due to the involvement of personal privacy (faces), these data are not publicly available.

## Abbreviations

The following abbreviations are used in this manuscript:

DL	Deep Learning
FR	Face Recognition
ABC	Automatic Border Control
GAN	Generative Adversarial Network
VGG	Visual Geometry Group
LFW	Labeled Faces in the Wild
FGSM	Fast Gradient Sign Method
BIM	Basic Iterative Method
PGD	Projected Gradient Descent
FAR	False Accept Rate
